# Food insecurity and cognitive function in older adults: findings from the longitudinal aging study in India

**DOI:** 10.1186/s12888-023-05118-8

**Published:** 2023-08-31

**Authors:** Alok Roy

**Affiliations:** Department of Geography, Krishnagar Government College, Krishnanagar, West Bengal 741101 India

**Keywords:** Food insecurity, Cognition, Older adults, LASI Wave 1

## Abstract

**Background:**

As we grow older, food insecurity (FI) may have an impact on our cognitive abilities. The study examines the association of FI with the cognitive function of older adults in India.

**Methods:**

We have used the data from the first wave of the Longitudinal Ageing Study of India (LASI), with a sample of 27,032 older adults aged 60 years and older. Bivariate analysis and linear regression models with clusters were applied to show the association. The cognitive performance tests include episodic memory, orientation, arithmetic function, executive function, and object naming.

**Results:**

The mean cognition was 24.2 (range 0–43), while 36.4%, 2.1%, and 6.4% experienced mild, moderate, and severe FI, respectively. After adjustment for potential confounders, mild (β = −0.18, 95% CI: −0.32, − 0.04) and severe (β = −0.52, 95% CI: −0.82, − 0.22) food insecurity was associated with poor overall cognitive performance. Domain-specific differences in cognition, such as memory, orientation, arithmetic function, executive function, and object naming, were also validated by the level of FI.

**Conclusion:**

The finding suggests that FI is associated with a poor level of cognition among older adults, highlighting the need for increasing the coverage and intervention strategies to address FI in India.

**Supplementary Information:**

The online version contains supplementary material available at 10.1186/s12888-023-05118-8.

## Background

According to Census 2011, India has 104 million older adults aged 60 and older, constituting 8.6% of the total population. The United Nations predicts that the percentage will rise even more, from 9.4 per cent in 2017 to 34.1 per cent by 2100 [1]. Food insecurity (FI) is defined as a reduction in the quality, variety, or desirability of one’s diet, as well as disrupted eating patterns with reduced food intake [2]. Moderate or severe FI at the global level has been slowly on the rise, from 22.6 per cent in 2014 to 26.6 per cent in 2019. Then in 2020, the year the COVID-19 pandemic spread across the globe, it rose nearly as much as in the previous five years combined, to 30.4 per cent. Thus, nearly one in three people in the world did not have access to adequate food in 2020–an increase of 320 million people in just one year, from 2.05 to 2.37 billion [3]. Despite being one of the world’s fastest-growing economies, India ranks 107th out of 121 countries in the 2022 Global Hunger Index, trailing many neighbouring countries and falling into the serious level of hunger category [4]. 6% of older adults aged 45 and older in India had to reduce the size of meals, 5% were hungry but did not eat because there was not enough food, and 4% did not eat for a full day because the food was unavailable in the past 12 months, according to the Longitudinal Ageing Study in India (LASI) reported [5].

FI is a significant social predictor of health, irrespective of other socioeconomic determinants [6]. Studies have shown that FI is associated with a variety of unfavourable health outcomes, including physical and mental health [7, 8]. Focusing on mental health problems, anxiety or poorer mental health [9], depression [8], suicidal ideation [10], and cognitive impairment [11, 12] have been related to FI.

Currently, there is increasing evidence linking FI to cognition [8, 13–15]. FI is regarded as one of the multiple factors that could expedite cognitive decline during old age [16]. Given that, it is well recognized that general cognitive abilities deteriorate with age [17, 18], the findings of links between FI and cognitive decline are especially concerning for older people because FI may put an additional strain on cognitive health. FI is associated with lower cognitive function across the life course, including at older ages [19]. FI is linked to a poor-quality diet, which includes eating fewer vegetables and fruits that are low in nutrients [20]. This decline in diet quality may indicate a quicker decline in cognitive function [21, 22]. FI may also increase stress [23], which has the potential to influence cognition [9]. In addition, there is mounting evidence linking FI to the decline in cognitive function in older adults [14, 24, 25].

Meanwhile, other factors may play a role in the variation in cognitive function including age [26, 27], sex [28–31], residence [11], marital status [32], education [33], wealth [34, 35], work history [33], self-rated health [36], BMI [37], ADLs/IADLs difficulties [38], morbidity [34, 39], childhood deprivation [40], alcohol consumption [41], smoking [42], social engagement [43] and physical activity [44], etc.

. The majority of investigations have concentrated on specific domains of cognition [12]. In addition, many studies have focused on proxy measures of cognitive function [11]. However, no studies have looked at the domain-specific association between cognition and FI in India. Thus, the current study aimed to investigate the prevalence of FI and assess whether FI is associated with poor cognitive function using data from a nationally representative sample of older adults aged 60 and older in India.

## Methods

### Study design and participants

**Fig. 1:**
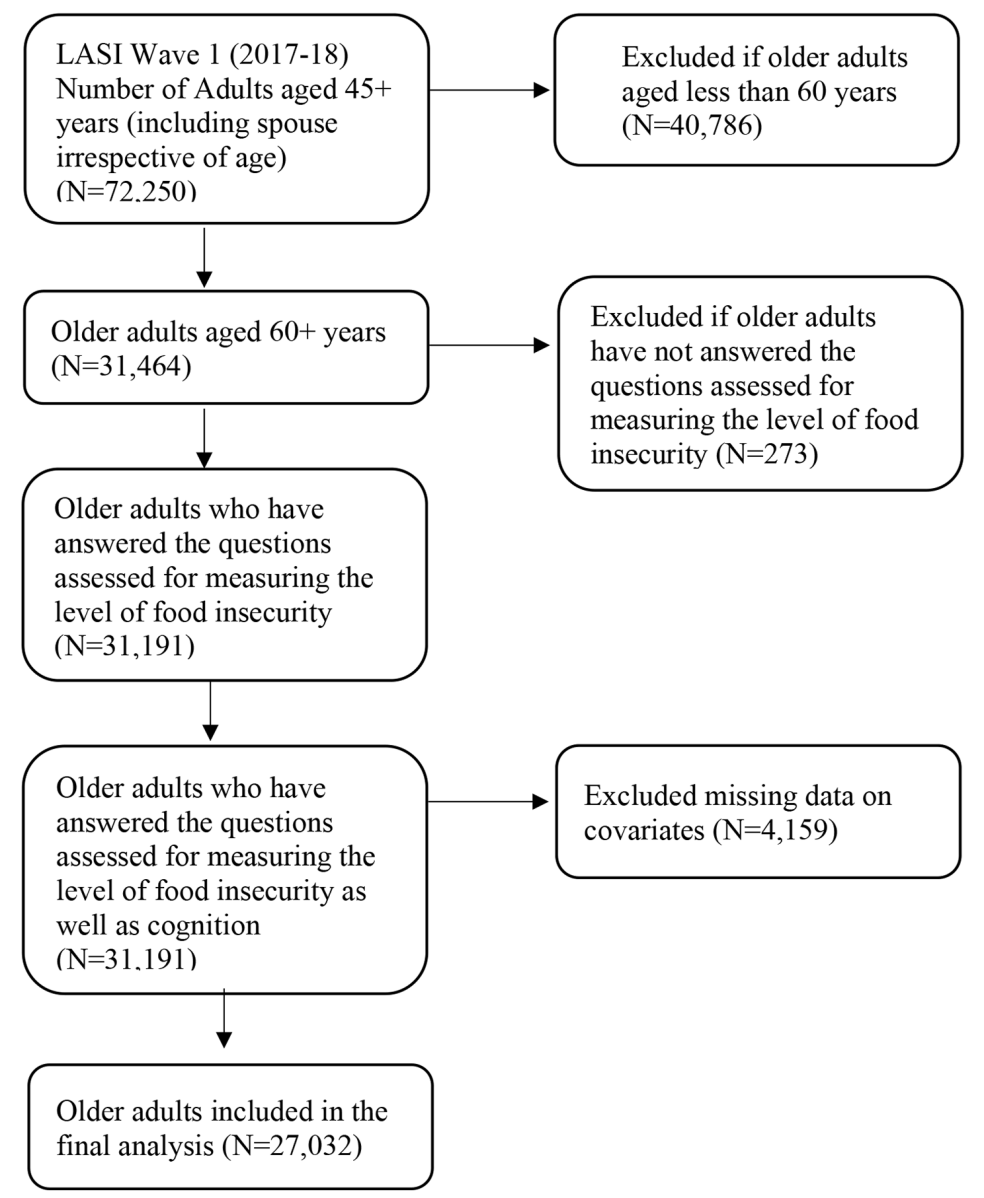
Sample selection flowchart from the first wave of Longitudinal Ageing Study in India (LASI Wave 1, 2017-18

Data from the LASI (Longitudinal Aging Study in India) wave 1 survey conducted in India between 2017 and 18 were analyzed. This dataset is publicly available to all interested researchers via the LASI website (https://iipsindia.ac.in/sites/default/files/LASI_DataRequestForm_0.pdf) upon request. The complete methodology, design, and data collection are easily available in the form of a report [5]. Briefly, LASI, harmonized with the Health and Retirement Study (HRS) is a nationwide comprehensive study on demographics, work, retirements, pensions, chronic health conditions, mental health, health care utilization, social and family networks, and biomarkers of older adults in India. It is the study of 72,250 older adults aged 45 or older, covering all 35 states (except Sikkim) and union territories (UTs). LASI adopted a multistage stratified area probability cluster sampling technique. For the household selection, three-stage sampling for rural areas and a four-stage sampling design for urban areas were followed. From 31,464 nationally representative older adults aged above 60 years in India, the present study involved 27,032 older adults after excluding respondents with missing values on FI (273), and all other covariates (4,159). A detailed sample selection procedure is demonstrated in Fig. 1.

### Measures

#### Cognition

The cognitive measures in the LASI were derived from the cognition module of the Health and Retirement Study (HRS), which includes five domains: memory, orientation, arithmetic, executive functioning, and object naming.

- Memory: For the memory test, the interviewer read out a list of 10 words, and respondents were asked to repeat the words immediately (ranges 0–10) and recall the same words after some time (ranges 0–10). So, the total word recall value ranges from 0 to 20.
- Orientation: Respondents were checked to see if they could state today’s date, month and year, day of the week, place of interview, name of the village, street number/colony name/ landmark/neighbourhood, and name of the district. 1 point is given for each correct answer and 0 points for a wrong one. The total score for orientation ranged from 0 to 8.
- Arithmetic function: Respondents were asked to count backwards as quickly as possible from the number 20 (range 0 to 2). Correctly counting received 2 points; counting with a mistake received 1 point. Those who could not count received 0 points. Respondents were asked to subtract 7 from 100 and repeat the same step five times (serial 7s) (range 0–5). The older adults were also given the task of solving mathematical operations (range 0–2). The total score ranged from 0 to 9.
- Executive function: Another domain (domain total range 0–4) in which respondents were given the task of paper folding (0 to 3) and pentagon drawing (0 to 1).
- Object naming: The interviewer points to a specific object and asks the respondent to name it. Two objects were pointed out, and one (1) point was given for each correct response.

A summary index or overall cognition score was calculated by summing up all the domains, and the value ranges from 0 to 43, implying that a higher cognition score indicates better cognitive ability and vice versa. A detailed description of the different cognitive domains measured in the LASI is presented in the supplementary file (Table S1). Please go through the LASI report [5] for more information.

#### Food Insecurity (FI)

The main explanatory factor in this study is the level of FI as measured by the Food Insecurity Experience Scale (FIES), a tool created by the Voices of the Hungry (VOH) initiative of the Food and Agricultural Organization (FAO). The scale’s eight well-tested, reliable questions can yield a consistent prevalence of FI across the settings [45]. Based on eight FIES items with dichotomous answers (Yes/No) asked the person, the severity level of FI was categorized as mild, moderate, and severe [46]. However, only four of those eight items, which were supposed to indicate the degree of FI a person had experienced in the 12 months before the survey, were asked of respondents in LASI. Given this constraint, we classified the four LASI questions according to Ballard et al. (2013)’s classification of the eight FIES items [46]:

- Mild food insecurity: “In the last 12 months, did you not eat enough food of your choice?“ Please exclude fasting or food-related restrictions due to religious or health-related reasons.
- Moderate food insecurity: “In the last 12 months, did you ever reduce the size of your meals or skip meals because there was not enough food in your household?“
- Severe food insecurity: “In the last 12 months, were you hungry but didn’t eat because there was not enough food at your household?“ or “Did you ever not eat for a whole day because there was not enough food at your household?“ Please exclude fasting or food-related restrictions for religious or health-related reasons.

#### Covariates

Past literature was used as a guide to select the control variables [11, 12, 26–44]. Socio-demographic covariates included age (continuous); sex (male, female); marital status (currently married, widowed, divorced, separated, or never married); education (no schooling, less than 5 years completed, 5–10 years, and 10 or more years); religion (Hindu, Muslim, and others); caste (scheduled caste [SC] or scheduled tribe [ST], other backward classes [OBC], and others); working status (never, worked, working); and the monthly per capita expenditure (MPCE) quintiles (i.e., from poorest to richest) [5].

Health-related behaviours, such as smoking history (yes, no, or never); drinking history (yes, no, or never); physical activity; and social activity were included in the study. Physical activity was a combination of three activities such as vigorous activity, including running, jogging, and swimming; moderate activity, including walking, cycling, and washing clothes; and other activities, including yoga and meditation, and each activity had five responses coded as 0 (hardly ever or never); 1 (one to three times a month); 2 (once a week); 3 (more than once a week); and 4 (every day). Social activity was the number of times respondents participated in various social activities per month, such as participating in community organizations, attending religious celebrations, or visiting relatives or friends [5].

Health-related variables include body mass index (BMI) based on measured weight and height (kg/m^2^); limitation in activities of daily living (ADL), which is defined as any limitations about dressing, walking across a room, bathing, eating, getting in and out of bed, and using the toilet; limitation in instrumental ADL (IADL), which is defined as any one of the limitations of food preparation, shopping, making phone calls, and taking medicine; chronic disease; and self-rated health (very good, good, and fair combined as good, whereas poor and very poor combined as poor). Chronic disease was assessed through nine (9) self-reported questions about whether they have ever been diagnosed by health experts with chronic diseases like diabetes, hypertension, cancer, chronic lung disease, heart disease, stroke, bone/joint diseases, dementia including Alzheimer’s disease, and cholesterol. Any of the one (1) self-reported chronic disease was categorized as yes, and otherwise no [5].

### Statistical approach

Bivariate analyses were carried out to assess the level of FI and mean cognition among older adults by their sociodemographic, health behaviour, health, and early life variables in India. Pearson’s chi-square test for categorical variables, ANOVA test, and Pearson’s correlation for continuous variables were used. The categorical variables were shown as percentages, and the continuous variables were shown as mean ± SD. The linear regression models with clusters were carried out to explore the associations between the level of FI and cognition. The equation of linear logistic regression can be written as follow:

$${\rm{y = }}\,{{\rm{\beta }}_{\rm{0}}}{\rm{ + }}{{\rm{\beta }}_{\rm{1}}}{{\rm{x}}_{\rm{1}}}{\rm{ + }}\,{{\rm{\beta }}_{\rm{2}}}{{\rm{x}}_{\rm{2}}}{\rm{ + }}\,{{\rm{\beta }}_{\rm{3}}}{{\rm{x}}_{\rm{3}}}{\rm{ + }}\,{{\rm{\beta }}_{\rm{4}}}{{\rm{x}}_{{\rm{4 \ldots \ldots }}..}}{{\rm{\beta }}_{\rm{n}}}{{\rm{x}}_{\rm{n}}}$$

Where y is the independent variables, β_0_ is the intercept, β_1,_ β_2,_ β_3,_ β_4,_ to β_n_ are the coefficients, and x_1,_ x_2,_ x_3,_ x_4,_ to x_n_ are the independent variables. Three separate models were employed. Model 1 was adjusted for age and sex. The regression coefficients in Model 2 were adjusted for sociodemographic covariates, and in Model 3, health-related behaviour, health, and early life covariates were adjusted. The results of the linear regression models were presented in terms of the beta coefficient and the 95% confidence interval (CI). The cognitive variables have undergone factor analysis, and the analysis reveals that only one factor is present. As highly correlated variables are combined into one factor during factor analysis (PCA or PCF), the eigenvalue corresponds to the sum of squared loadings for a factor, allowing the findings of multivariable relationships. The variance explained by an eigenvalue’s variance is 100% if there is a common factor that explains the variance of all variables. In the supplemental file, a thorough factor analysis was presented. (see Table S2, S3, S4, and Fig. S1 for details, available online). STATA software (SE), version 16, was used for all statistical analyses.

## Results

### Socio-demographic profile of older adults

Table 1 displays the sample characteristics, the level of FI, and the mean overall cognition among older adults aged 60 + years by sociodemographic, health, and early life characteristics. A total of 27,032 older adults were included, including 13,050 (47.4%) males and 13,982 (52.6%) females. Two in every five (44.9%) older adults in India have experienced different levels of FI i.e., mild (36.4%), moderate (2.1%), and severe (6.4%). About 71.8% were from rural areas; 62.4% were married; 46.8% were from other backward classes (OBC); 83.9% were from the Hindu religion; more than half (56.5%) had no education; 31.9% were working; 40.6% had a smoking; and 15.0% had a drinking history. The mean physical activity, social activity, and BMI were 3.8 ± 3.2, 9.7 ± 7.8, and 22.1 ± 4.7 respectively. 23.3% of the older adults reported their health as poor, 21.6% reported difficulty in ADL, and 47.2% reported difficulty in IADL. One in every two (52.6%) older adults were suffering from a chronic disease. Further details are shown in Table 1.

**Table 1:** Percentage distribution of older adults (60 + years) by the level of food insecurity and the mean cognition by sociodemographic, health behavior, health, and early life variables in India, LASI Wave-1 (2017-18)

	Level of food insecurity				p-value	Cognition^*^	p-value	Sample size (%) or mean (SD)
	Food secure	Mild	Moderate	Severe				
**Level of food insecurity**								
Food secure	-	-	-	-		24.9	< 0.001	15,127(55.0)
Mild	-	-	-	-		23.6		9,978(36.4)
Moderate	-	-	-	-		23.2		564(2.1)
Severe	-	-	-	-		22.0		1,363(6.4)
**Age**	68.8 (± 7.2)	69.0 (± 7.4)	69.0 (± 7.6)	68.3 (± 7.3)	< 0.001	-0.241	< 0.001	68.6(± 7.3)
**Sex**								
Male	56.2	35.8	1.9	6.1	< 0.001	26.7	0.003	13,050(47.4)
Female	54.3	37.0	2.2	6.6		22.0		13,982(52.6)
**Residence**								
Rural	55.1	36.8	2.3	5.8	< 0.001	23.1	< 0.001	18,151(71.8)
Urban	57.8	37.1	1.7	3.5		27.0		8,881(28.2)
**Marital status**								
Married	56.9	35.7	1.9	5.6	< 0.001	25.6	< 0.001	17,334(62.4)
Widow/div/sep/never	52.5	37.5	2.3	7.7		21.9		9,698(37.6)
**Caste**								
SC/ST	50.2	38.1	2.5	9.2	< 0.001	22.1	< 0.001	9,171(27.7)
OBC	55.9	36.6	1.9	5.6		24.5		10,642(46.8)
Others	59.4	34.2	1.8	4.6		25.9		7,219(25.5)
**Religion**								
Hindu	55.3	36.5	2.0	6.1	< 0.001	24.3	0.006	20,131(83.9)
Muslim	54.8	34.8	2.4	8.0		23.9		2,906(9.7)
Others	54.1	37.0	2.1	6.8		23.9		3,995(6.4)
**Education**								
No Schooling	52.6	37.3	2.1	8.0	< 0.001	20.7	< 0.001	14,448(56.5)
Less than 5 years	53.6	36.8	3.1	6.6		25.3		3,335(11.7)
5–9 years	58.0	35.2	1.8	4.9		28.7		5,244(18.0)
10 or more	63.7	33.7	1.1	1.4		31.8		4,005(13.9)
**MPCE quintile**								
Poorest	48.5	40.3	2.0	9.2	< 0.001	22.7	< 0.001	5,581(21.7)
Poorer	54.9	36.2	2.5	6.5		23.5		5,592(21.6)
Middle	56.7	35.8	1.8	5.6		24.5		5,570(20.9)
Richer	59.1	33.8	1.6	5.5		24.9		5,319(19.4)
Richest	58.0	35.2	2.3	4.4		25.9		4,970(16.4)
**Working status**								
Never	53.9	40.2	1.6	4.3	< 0.001	23.0	< 0.001	7,386(26.1)
Worked	55.8	35.4	2.3	6.6		24.1		11,353(42.0)
Working	55.6	34.6	2.1	7.7		25.4		8,293(31.9)
**Smoking**								
Yes	56.4	35.7	2.1	5.8	< 0.001	24.4	< 0.001	10,694(40.6)
No	55.7	37.7	2.1	4.6		24.1		16,338(59.4)
**Drinking**								
Yes	57.9	33.3	2.2	6.8	< 0.001	25.1	< 0.001	4,782(15.0)
No	54.8	36.9	2.0	6.3		24.0		22,250(85.0)
**Physical activity** ^@^	4.0 (± 3.1)	3.5 (± 3.1)	3.7 (± 3.2)	3.7 (± 3.2)	< 0.001	0.182	< 0.001	3.8 (± 3.2)
**Social activity** ^#^	9.6 (± 7.8)	8.9 (± 8.0)	7.9 (± 7.6)	6.5 (± 6.9)	< 0.001	0.446	< 0.001	9.7 (± 7.8)
**BMI (kg/m** ^**2**^ **)**	22.0 (± 4.8)	21.6 (± 4.8)	20.7 (± 4.2)	20.4 (± 4.3)	< 0.001	0.265	< 0.001	22.1 (± 4.7)
**SRH**								
Good/very good	60.4	33.7	2.0	3.9	< 0.001	25.5	< 0.001	9,147(31.5)
Fair	55.4	36.6	1.9	6.1		24.2		11,816(45.2)
Poor/very poor	47.9	39.5	2.4	10.2		22.4		6,069(23.3)
**ADL limitation**								
Yes	52.1	34.8	3.0	10.0	< 0.001	22.0	< 0.001	5,231(21.6)
No	56.1	36.8	1.8	5.4		24.8		21,801(78.5)
**IADL limitation**								
Yes	54.6	34.6	2.6	8.2	< 0.001	22.1	< 0.001	11,613(47.2)
No	55.8	38.0	1.6	4.7		26.0		15,419(52.8)
**Chronic disease**								
Yes	54.8	37.0	2.1	6.1	< 0.001	24.7	0.173	14,440(52.6)
No	55.7	35.7	2.0	6.6		23.7		12,592(47.4)
**Childhood SRH**								
Very good/good	57.7	35.5	2.0	4.8	< 0.001	24.3	< 0.001	23,960(87.8)
Fair	41.4	49.1	2.6	6.9		23.3		2,662(10.5)
Poor/very poor	47.6	39.3	5.1	8.1		23.5		410(1.7)
**Childhood finance**								
Financially well	61.7	31.9	1.7	4.7	< 0.001	26.1	< 0.001	2,087(8.4)
Average	54.4	39.1	1.7	4.8		25.1		14,067(48.3)
Poor	54.9	34.2	2.5	8.5		22.8		10,878(43.3)
**All sample**	55.2	36.4	2.1	6.4		24.2		27,032(100)

Data are shown as mean ± SD or weighted percentage; p-value derived from the ANOVA test for continuous variables, the chi-square test for categorical variables, and Pearson’s correlation for continuous variables (both); * 0 is the worst and 43 is the best cognition; BMI: body mass index; SC: Scheduled caste; ST: Scheduled tribe; OBC: Other backward class; MPCE: monthly per capita expenditure; ADL/IADL: Activity/Instrumental activity of daily living; The sample size may not correspond to the percentages because of weighted cases; ^@^range: 0–12; ^#^range: 0–66

Table 1 also shows that FI (i.e., mild, moderate, and severe) was more prevalent among females, in rural areas, among widows/divorced/separated/never married, among SC/ST communities, and Muslim religious communities. With increasing education and MPCE quintiles, a decline in severe FI was worth noting. Meanwhile, severe FI was prevalent among working older adults and older adults with a history of smoking. The mean physical activity, social activity, and BMI decline along with the increasing severity of FI. Poor SRH, ADL limitation, IADL limitation, chronic disease, poor childhood SRH, and poor childhood finance were positively associated with the severity level of FI.

It is also apparent that the mean cognition differed significantly between males and females, older adults living in rural and urban areas, married and widows/divorcees/separated/never married, and across castes, with lower cognition prevalent in SC and ST communities. While there was no significant difference in cognition across religions, it is significantly comparable across education levels and the MPCE quintiles. The significant difference in cognition also exists across working status, SRH, ADL limitation, IADL limitation, chronic disease, childhood SRH, and childhood finance. Positive correlations were found between physical activity, social activity, BMI, and cognition. The mean cognition of the study sample was 24.2 (range 0–43).

### Domain-wise mean cognition by food security

The mean scores for each cognition domain by the level of FI are shown in Table 2. It displays an unweighted mean cognition score, with higher scores indicating better cognition and vice versa. The mean cognition scores were observed to be lower among those who were food insecure (i.e., mild, moderate, and severe) than those with food security in each domain. The cognition declines with the severity level of FI. The mean cognition on the memory, orientation, arithmetic function, executive function, and object naming tests was 8.5, 6.1, 3.4, 2.0, and 1.9, respectively, among older adults with severe FI, and the cognition scores were lower than all other FI categories. The overall mean cognition score was lowest among severely food-insecure older adults (mean cognition: 21.9) and highest among food-secure older adults (mean cognition: 24.9).

**Table 2:** Mean scores on cognitive function by the level of food insecurity among 27,032 older adults in India: LASI wave 1, 2017–18

Domain	Range	Food secure (15,127)	Mild food insecurity (9,978)	Moderate food insecurity (564)	Severe food insecurity (1,363)	p-value*
Memory	0–20	9.4(3.8)	9.3(3.7)	9.0(3.7)	8.5(3.6)	< 0.001
Orientation	0–8	6.7(1.5)	6.6(1.6)	6.4(1.5)	6.1(1.6)	< 0.001
Arithmetic function	0–9	4.5(3.0)	4.2(3.0)	3.7(2.8)	3.4(2.8)	< 0.001
Executive function	0–4	2.4(1.1)	2.2(1.1)	2.1(1.0)	2.0(1.0)	< 0.001
Object naming	0–2	2.0(0.2)	1.9(0.4)	1.9(0.4)	1.9(0.4)	< 0.001
Overall cognition	0–43	24.9 (7.3)	24.3(7.6)	23.2(6.9)	21.9(7.0)	< 0.001

Values are means ± SD *P-value from the ANOVA test

Memory: Immediate & delayed word recall; Orientation: Place & time; Arithmetic function: Backward counting, serial 7 & computation; Executive function: paper folding & pentagon drawing

**Fig. 2:**
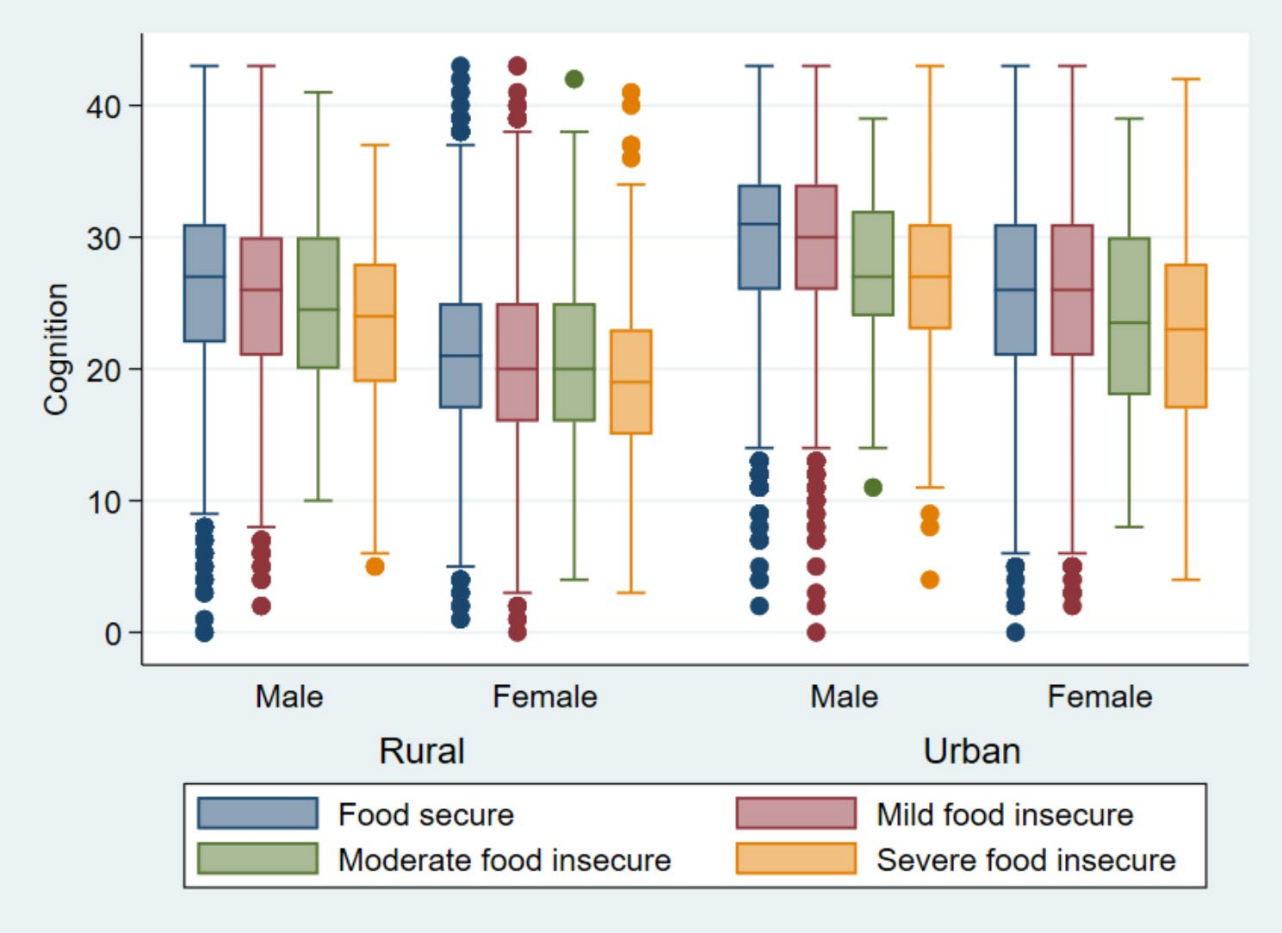
The mean cognition by sex and residence across the level of food insecurity

Figure 2 depicts the mean cognition of older adults by sex and residence across the level of FI. The mean cognition was higher among urban males and lower among rural females in all the FI categories. Similarly, the mean cognition is lowest among older adults with severe FI and highest among older adults with food security across the residence and sex (rural male, rural female, urban male, urban female).

### Association between the level of FI and cognition among older adults

The results of linear regression for the associations between the level of FI and cognition are presented in Table 3. β coefficient and 95% confidence interval (CI) were computed to comprehend the association and its significance. There were three different models utilized. After adjusting for age and sex, the levels of FI (i.e., mild, moderate, and severe) were significantly associated with lower cognition for each domain of the cognition test and the overall cognition in model I. After adjusting for all sociodemographic characteristics in model II, mild (β = −0.27, 95% CI: −0.41, − 0.13), moderate (β = −0.48, 95% CI: −0.95, − 0.01), and severe (β = −1.05, 95% CI: −1.36, − 0.74) FI were significantly associated with lower overall cognition. After adjusting for all sociodemographic, health behaviour, health, and early life characteristics in model III, the result remained significant, where mild (β = −0.18, 95% CI: −0.32, − 0.04) and severe (β = −0.52, 95% CI: −0.82, − 0.22) FI were related to lower overall cognition. Associations were also observed between severe FI and orientation (β = −0.18, 95% CI: −0.25, − 0.11), arithmetic function (β = −0.09, 95% CI: −0.20, − 0.03), executive function (β = −0.14, 95% CI: −0.19, − 0.08) and object naming (β = −0.03, 95% CI: −0.05, − 0.02). The association between moderate FI and each domain of cognition was no longer significant after further adjustment of additional covariates, except executive function (β = −0.10, 95% CI: −0.18, − 0.02) in model III.

**Table 3:** Associations between the level of food insecurity and cognitive function among older adults, LASI wave 1, 2017-18

Level of food insecurity	Model I		Model-II		Model-III	
	β (95%CI)	p-value	β (95%CI)	p-value	β (95%CI)	p-value
**Memory**						
Mild	-0.08(-0.17,0.02)	0.106	0.01(-0.07,0.10)	0.760	0.05(-0.03,0.14)	0.239
Moderate	-0.35(-0.66,-0.05)	0.024	-0.02(-0.31,0.27)	0.905	0.13(-0.15,0.42)	0.361
Severe	-0.87(-1.07,-0.67)	< 0.001	-0.33(-0.53,-0.14)	0.001	-0.08(-0.27,0.11)	0.412
**Orientation**						
Mild	-0.11(-0.15,-0.08)	< 0.001	-0.07(-0.11,-0.04)	< 0.001	-0.07(-0.10,-0.04)	< 0.001
Moderate	-0.28(-0.40,-0.16)	< 0.001	-0.10(-0.21,0.01)	0.076	-0.03(-0.14,0.08)	0.585
Severe	-0.58(-0.66,-0.50)	< 0.001	-0.28(-0.35,-0.21)	< 0.001	-0.18(-0.25,-0.11)	< 0.001
**Arithmetic function**						
Mild	-0.22(-0.29,-0.15)	< 0.001	-0.08(-0.14,-0.03)	0.002	-0.06(-0.11,-0.01)	0.027
Moderate	-0.67(-0.90,-0.44)	< 0.001	-0.19(-0.37,-0.01)	0.036	-0.11(-0.29,0.06)	0.202
Severe	-0.95(-1.10,-0.80)	< 0.001	-0.20(-0.32,-0.08)	0.001	-0.09(-0.20,0.03)	0.153
**Executive function**						
Mild	-0.13(-0.16,-0.10)	< 0.001	-0.10(-0.12,-0.07)	< 0.001	-0.08(-0.10,-0.05)	< 0.001
Moderate	-0.25(-0.34,-0.17)	< 0.001	-0.14(-0.23,-0.06)	0.001	-0.10(-0.18,-0.02)	0.013
Severe	-0.36(-0.42,-0.30)	< 0.001	-0.20(-0.25,-0.14)	< 0.001	-0.14(-0.19,-0.08)	< 0.001
**Object naming**						
Mild	-0.03(-0.04,-0.02)	< 0.001	-0.03(-0.04,-0.02)	< 0.001	-0.03(-0.03,-0.02)	< 0.001
Moderate	-0.04(-0.06,-0.01)	0.008	-0.03(-0.06,0.00)	0.027	-0.02(-0.05,0.01)	0.117
Severe	-0.05(-0.07,-0.04)	< 0.001	-0.04(-0.06,-0.03)	< 0.001	-0.03(-0.05,-0.02)	< 0.001
**Overall Cognition**						
Mild	-0.57(-0.74,-0.40)	< 0.001	-0.27(-0.41,-0.13)	< 0.001	-0.18(-0.32,-0.04)	0.010
Moderate	-1.60(-2.17,-1.03)	< 0.001	-0.48(-0.95,-0.01)	0.044	-0.13(-0.59,0.32)	0.563
Severe	-2.81(-3.19,-2.44)	< 0.001	-1.05(-1.36,-0.74)	< 0.001	-0.52(-0.82,-0.21)	0.001

**Note**: Authors’ own calculation-using data from the 2017-18 Longitudinal Aging Study in India (LASI wave 1)

Model 1 was adjusted for age and sex. Regression coefficients in Model 2 were adjusted for all covariates in Model 1 plus sex. residence, marital status, caste, religion, education, MPCE quintiles, and working status. Regression coefficients in Model 3 were adjusted for all covariates in Model 2 plus smoking, drinking, physical activity, social activity, body mass index (BMI) (Kg/m2), self-rated health (SRH), activities of daily living (ADL), instrumental activities of daily living (IADL), chronic disease, childhood SRH, and childhood financial condition.

Reference food insecurity category: food security; CI: confidence interval

## Discussion

In this study, we investigated the prevalence of FI among older Indian adults and the association between cognition and FI. The mean cognition of the study sample was 24.2. In this national sample of older adults, about two in every five older adults (44.9%) in India are food insecure. After adjustment for potential confounders, mild and severe FI was inversely associated with cognition. Differences in general cognition, such as memory, orientation, arithmetic function, executive function, and object naming were also validated by the level of FI. To the best of our knowledge, this is the first study to specifically examine the relationship between FI and cognition covering all the domains. Our findings are in line with previous studies that have examined the association between FI and cognition in India [11, 12], although these studies were on specific domains [12] or proxy measures of cognitive impairment [11] and not overall cognition. A community-based cross-sectional study of 31,464 Indians aged 60 + years found that those who were food secure were less likely to have word recall and computational problems than those who were insecure [12]. Another cross-sectional study using data from the LASI has highlighted that older adults who did not have enough food of their choice had significantly higher odds of suffering from cognitive impairment in comparison to their counterparts. Similarly, the older adults who were hungry but did not eat were 30% more likely to suffer from cognitive impairment in comparison to their counterparts [11].

In addition, the findings of the study corroborate the findings from the USA [14], India [11, 12], South Africa [24], Malaysia [47], etc. FI was found to be negatively correlated with overall cognitive performance, as measured by the MMSE score, in a sample of 1,358 Puerto Ricans living in Massachusetts [6]. In a cross-sectional community-based study on individuals aged ≥ 50 years in South Africa (2007–08), FI was associated with mild cognitive impairments (MCI), among older adults [24]. Another study has shown that compared to those who were fully food secure, those who were marginally food secure, food insecure without hunger, and food insecure with hunger had significantly lower cognitive function [15].

Additionally, it is evident from the supplementary file (Table S5, S6, S7, S8, S9, S10) that cognitive functions decline with age [26, 27]. Gender differences in cognitive function, favouring males, were very prevalent in earlier studies [28–31]. Studies also found lower cognitive function among the rural elderly [11]. According to a study on marital status and health-related outcomes among older adults in India, widowhood may be a risk factor for reduced cognitive ability in women [32]. The odds of cognitive impairment were higher among uneducated older adults and older adults in the lowest wealth quintile [33, 34]. The study reveals caste differences in cognitive function in India. Poorer elderly people have lower cognitive function; however, wealth disparities diminish with age [35]. Older women without current or prior work history report lower odds of cognitive impairment compared to their peers in the labour force [33].

The previous findings also show that poor self-rated health (SRH) is associated with lower cognitive function [36]. Nutritional status also determines the cognitive performance of older adults. An underweight BMI is associated with poor cognition [37]. The cognitive function declines along with the increase of ADLs/IADLs difficulties [38]. Chronic morbidities were negatively associated with cognition [34, 39]. Childhood deprivation predicts late-life cognitive impairment in Indian older adults [40].

Apart from that, alcohol consumption did have a negative association with cognitive function [41]. Older adults who smoked tobacco or drank alcohol had a significantly higher risk of cognitive impairment than older adults who did not smoke or drink [42]. The previous literature highlighted the positive association of social engagement with cognitive function [43]. A one-point increase in the social activity score was associated with a 47% decrease in the rate of decline in global cognitive function. Older adults who engage in frequent physical activity have greater cognitive functioning than older adults without physical activity [44]. Childhood deprivations predict later-life cognitive impairment among older adults [40]. There have been limited contemporary studies examining this relationship among older populations in low- and middle-income countries (LMICs) settings, despite the association between FI and psychological distress, which is a growing public health concern.

Although the exact mechanisms relating FI to MCI are unclear, there are a couple of hypotheses. Inadequate nutritional intake and higher psychological stress are potential reasons for the associations. FI is a situation that is naturally stressful and has been linked to poor mental health [23]. Furthermore, FI frequently has an impact on diet quality [48], as people switch to less nutritious, more affordable foods when food is scarce. Poor diet has been linked to an increased risk of cognitive decline. Key vitamins, for instance, have all been demonstrated to have protective effects against dementia [49]. The study’s large sample size and utilization of nationally representative data are among its strengths. To avoid ceiling effects in any domain of the cognitive test, an overall cognitive function score is calculated. The most recent data available gives an overview of the situation among elderly individuals in India. Another strength of the study is the ability to adjust for the effects of various sociodemographic and health covariates in the model. However, some limitations should be taken into consideration when interpreting the findings. Furthermore, our measure of FI was based on four questions and did not represent a comprehensive measure of FI. The direction of causation cannot be inferred because this was a cross-sectional study. Therefore, we could not measure the changes in cognition due to changes in FI. For example, people with cognitive impairments may have difficulty utilizing social safety net services, and this might have led to FI. Therefore, there may be a bidirectional relationship between poor mental health and FI [50]. Even though we took into account many potential confounders, we cannot rule out the possibility of residual confounding driven by unidentified factors. FI in early life can significantly contribute to dementia in later life [47]. Lastly, the mediation or indirect effect of many confounders cannot be ignored.

## Conclusion

In conclusion, the findings revealed the high prevalence of FI among Indian adults, which was strongly associated with lower cognitive performance. To reduce FI and maintain cognitive health in these disadvantaged populations, programs, and policies that increase the availability of nutritious food are required. In addition, expanding the public distribution system’s (PDS) reach could aid in reducing India’s FI. Although this study suggests that FI may lead to poor cognition, future longitudinal studies are needed to assess causality and the benefits of solving FI as a preventive approach for poor cognition and cognitive impairments.

### Electronic supplementary material

Below is the link to the electronic supplementary material.

Supplementary Material 1: Tables S1, S2, S3, S4, S5, S6, S7, S8, S9, S10 and Fig. S1.

## Data Availability

The LASI Wave-1 data was collected by the nodal institution International Institute for Population Sciences (IIPS), Mumbai on behalf of the Ministry of Health and Family Welfare, Government of India. The LASI Wave-1 data is publicly available to the researchers and policymakers upon formal request to the International Institute for Population Sciences for access through https://iipsindia.ac.in/sites/default/files/LASI_DataRequestForm_0.pdf.

## Abbreviations

BMI: Body mass index
SC: Scheduled caste
ST: Scheduled tribe
OBC: Other backward class
CI: Confidence Interval
MPCE: Monthly per capita consumption Expenditure
ADL: Activities of daily living
IADL: Instrumental activities of daily living
SRH: Self-rated health
CSED: Centre of Epidemiologic Studies-Depression
