# Supplementary material for: Food insecurity and cognitive function in older adults: findings from the longitudinal aging study in India
Source: BMC Psychiatry. 2023 Aug 31;23:640. doi: 10.1186/s12888-023-05118-8 (PMC10472592; doi:10.1186/s12888-023-05118-8)
Supplement: Supplementary file 1 — Supplementary Material 1: Tables S1, S2, S3, S4, S5, S6, S7, S8, S9, S10 and Fig. S1. [file 12888_2023_5118_MOESM1_ESM.docx]

**Table S1: Description of domain-wise cognitive measures**

| **Domain** | **Measure** | **Measurement** | **Range** |
| --- | --- | --- | --- |
| **Memory** | Immediate word  recall | The interviewer read out a list of 10 words and respondents were asked to repeat the words. | 0-10 |
|  | Delayed word recall | Respondents were asked to recall the same words read out for immediate recall after some time. | 0-10 |
|  | Total word recall | Sum of immediate and delayed word recall | 0-20 |
| **Orientation** | Time | Respondents were asked to state today’s date, month and year, and day of the week. For each question, the score was 0 or 1. Correct responses received 1 point, and incorrect responses received 0. The total score for time was 0-4. | 0-4 |
|  | Place | Orientation towards place was captured based on the place of interview, name of the village, street number/colony name/ landmark/neighbourhood, and name of the district.  Each correct response scored 1 point. The total score ranged from 0-4. | 0-4 |
| **Retrieval fluency** | Verbal fluency* | Participants were asked to mention as many names of animals/birds as possible in one minute. | 0-61 |
| **Arithmetic function** | Backward counting | Respondents were asked to count backward as quickly as possible from the number 20. The respondents were asked to stop after correctly counting backward from 20 to 11 or from 19 to 10. Correct counting received 2 points; counts with a mistake received 1 point. Those who could not count received 0 points. | 0-2 |
|  | Serial 7 | Respondents were asked to subtract seven from 100 in the first step and asked to continue subtracting seven from the previous number in each subsequent step for five times. Each correct response received 1 point. | 0-5 |
|  | Computation | This test involved the mathematical operation of division. Respondents were asked to compute the net sale price of a product after considering a discount sale of half of the original price. | 0-2 |
| **Executive function: 0-4** | Executive (paper folding) | This is a three-stage command task. The respondents were instructed to take a piece of paper from the interviewer, turn it over, fold it in half, and give it back to the interviewer. Three points were given if each task was completed successfully. | 0-3 |
|  | Pentagon drawing | Visio-construction is the ability to coordinate fine motor skills with visuospatial abilities, usually by reproducing geometric figures. Respondents were asked to copy two overlapping pentagons and scored 1 point for a correct drawing. | 0-1 |
| **Object naming: 0-2** |  | The interviewer points to a specific object and asks the respondent to name it. Two objects were pointed out and 1 point was given for each correct response. | 0-2 |
| **Cognition** | Composite cognitive index | The combined score of memory (total word recall), orientation, arithmetic function, executive function, and object naming. | 0-43 |

Source: Longitudinal Aging Study in India (LASI) Wave 1(2017-18) Report *The verbal fluency score is not included in the composite cognitive index.

| **Table S2: Correlation matrix of cognitive variables** | | | | | |
| --- | --- | --- | --- | --- | --- |
|  | Memory | Orientation | Arithmetic function | Executive function | Object Naming |
| Memory | 1.000 |  |  |  |  |
| Orientation | 0.424 | 1.000 |  |  |  |
| Arithmetic function | 0.436 | 0.587 | 1.000 |  |  |
| Executive function | 0.303 | 0.371 | 0.453 | 1.000 |  |
| Object Naming | 0.116 | 0.194 | 0.136 | 0.1759 | 1.000 |
| *Note: cognitive variables are not highly correlated* | | | | | |

| **Table S3: Principal component factors (PCF) (n=27,032)** | | | | |
| --- | --- | --- | --- | --- |
| Factor | Eigenvalue | Difference | Proportion | Cumulative |
| **Factor1** | **2.3695** | **1.4291** | **0.4739** | **0.4739** |
| Factor2 | 0.9404 | 0.2427 | 0.1881 | 0.6620 |
| Factor3 | 0.6977 | 0.1028 | 0.1395 | 0.8015 |
| Factor4 | 0.5949 | 0.1974 | 0.1190 | 0.9205 |
| Factor5 | 0.3975 | . | 0.0795 | 1.0000 |
| *Note: we are looking for Eigenvalue above 1.0* | | | | |

| **Table S4: Factor loadings (pattern matrix) and unique variances** | | | |
| --- | --- | --- | --- |
| **Variable** | **Factor1** | **Uniqueness** | **Communality** |
| Memory | 0.6406 | 0.5897 | 0.4103 |
| Orientation | 0.782 | 0.3885 | 0.6115 |
| Arithmetic function | 0.8195 | 0.3283 | 0.6717 |
| Executive function | 0.6838 | 0.5325 | 0.4675 |
| Object Naming | 0.2855 | 0.9185 | 0.0815 |
| *Based on Eigen value exceeding 1.0, only 1 factor has been extracted; Uniqueness is the percentage of variance for the variable that is explained by the common factor. Values more than 0.6 are generally high, which means the variable is not well explained by the factors; communality=1-uniqueness.* | | | |


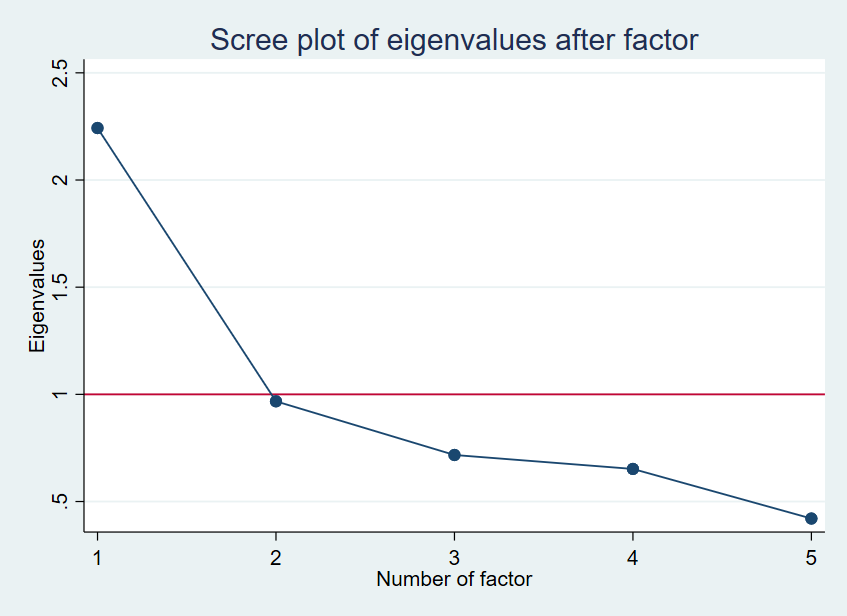


**Fig. S1: Scree plot of eigenvalues after factor**

| **Table S5: Associations between the level of food insecurity and memory among older adults, LASI wave 1, 2017-18** | | | | | | |
| --- | --- | --- | --- | --- | --- | --- |
|  | **Model I** | | **Model-II** | | **Model-III** | |
|  | **β (95%CI)** | **p-value** | **β (95%CI)** | **p-value** | **β (95%CI)** | **p-value** |
| Food insecurity (ref: food secure) | | | |  |  |  |
| Mild food  insecure | -0.08(-0.17,0.02) | 0.106 | 0.01(-0.07,0.10) | 0.760 | 0.05(-0.03,0.14) | 0.239 |
| Moderate  food insecure | -0.35(-0.66,-0.05) | 0.024 | -0.02(-0.31,0.27) | 0.905 | 0.13(-0.15,0.42) | 0.361 |
| Severe food  insecure | -0.87(-1.07,-0.67) | 0.000 | -0.33(-0.53,-0.14) | 0.001 | -0.08(-0.27,0.11) | 0.412 |
| Age | -0.11(-0.12,-0.10) | 0.000 | -0.09(-0.10,-0.08) | 0.000 | -0.07(-0.08,-0.06) | 0.000 |
| Female | -0.85(-0.98,-0.72) | 0.000 | -0.09(-0.26,0.08) | 0.275 | -0.27(-0.46,-0.07) | 0.008 |
| Rural |  |  | -0.62(-0.91,-0.34) | 0.000 | -0.30(-0.57,-0.02) | 0.034 |
| Widow/div/Sep/others | | | -0.35(-0.47,-0.23) | 0.000 | -0.25(-0.37,-0.12) | 0.000 |
| Caste (ref: none) | | |  |  |  |  |
| SCs/STs |  |  | -0.41(-0.69,-0.13) | 0.006 | -0.26(-0.53,0.01) | 0.059 |
| OBCs |  |  | 0.05(-0.21,0.32) | 0.685 | 0.11(-0.16,0.39) | 0.402 |
| Religion (ref: Hindu) | | | 0.00(0.00,0.00) |  | 0.00(0.00,0.00) |  |
| Muslim |  |  | 0.03(-0.23,0.28) | 0.830 | -0.04(-0.36,0.29) | 0.812 |
| Others |  |  | 0.00(-0.35,0.34) | 0.993 | -0.25(-0.65,0.14) | 0.194 |
| Education (ref: no schooling) | | |  |  |  |  |
| Standard 1-4 | |  | 0.69(0.45,0.92) | 0.000 | 0.47(0.24,0.70) | 0.000 |
| Standard 5-9 | |  | 1.36(1.16,1.57) | 0.000 | 0.98(0.79,1.18) | 0.000 |
| Standard 10 and above | |  | 2.54(2.27,2.80) | 0.000 | 1.76(1.50,2.02) | 0.000 |
| MPCE quintile (ref: poorest) | | |  |  |  |  |
| Poorer |  |  | 0.05(-0.14,0.25) | 0.591 | -0.03(-0.20,0.14) | 0.733 |
| Middle |  |  | 0.25(-0.02,0.52) | 0.071 | 0.08(-0.17,0.32) | 0.537 |
| Richer |  |  | 0.35(0.05,0.65) | 0.023 | 0.14(-0.12,0.41) | 0.279 |
| Richest |  |  | 0.53(0.19,0.87) | 0.003 | 0.25(-0.03,0.53) | 0.083 |
| Working status (ref: working) | | | |  |  |  |
| Not working | |  | -0.06(-0.28,0.17) | 0.624 | -0.02(-0.24,0.19) | 0.824 |
| Working |  |  | 0.10(-0.20,0.41) | 0.498 | -0.08(-0.37,0.21) | 0.580 |
| Smoking: no | |  |  |  | -0.02(-0.20,0.17) | 0.866 |
| Drinking: no | |  |  |  | 0.45(0.14,0.77) | 0.006 |
| Physical activity | |  |  |  | 0.08(0.05,0.12) | 0.000 |
| Social activity | |  |  |  | 0.05(0.04,0.07) | 0.000 |
| BMI |  |  |  |  | 0.05(0.04,0.07) | 0.000 |
| SRH (ref: good/very good) | | |  |  |  |  |
| Fair |  |  |  |  | -0.12(-0.34,0.10) | 0.272 |
| Poor/very poor | |  |  |  | -0.51(-0.78,-0.23) | 0.001 |
| ADL limitations | |  |  |  | -0.31(-0.51,-0.11) | 0.003 |
| IADL limitations | |  |  |  | -0.24(-0.43,-0.04) | 0.019 |
| Chronic disease: yes | |  |  |  | 0.08(-0.07,0.24) | 0.288 |
| Childhood SRH (ref: good/very good) | | | |  |  |  |
| Fair |  |  |  |  | -0.06(-0.32,0.19) | 0.606 |
| Poor/very poor | |  |  |  | 0.19(-0.18,0.55) | 0.314 |
| Childhood finance (ref: financially well) | | | |  |  |  |
| Average |  |  |  |  | 0.17(-0.06,0.39) | 0.138 |
| Poor |  |  |  |  | -0.17(-0.43,0.09) | 0.195 |

| **Table S6: Associations between the level of food insecurity and orientation among older adults, LASI wave 1, 2017-18** | | | | | | |
| --- | --- | --- | --- | --- | --- | --- |
|  | **Model I** | | **Model-II** | | **Model-III** | |
|  | **β (95%CI)** | **p-value** | **β (95%CI)** | **p-value** | **β (95%CI)** | **p-value** |
| Food insecurity (ref: food secure) | | |  |  |  |  |
| Mild food  insecure | -0.11(-0.15,-0.08) | 0.000 | -0.07(-0.11,-0.04) | 0.000 | -0.07(-0.10,-0.04) | 0.000 |
| Moderate  food insecure | -0.28(-0.40,-0.16) | 0.000 | -0.10(-0.21,0.01) | 0.076 | -0.03(-0.14,0.08) | 0.585 |
| Severe  food insecure | -0.58(-0.66,-0.50) | 0.000 | -0.28(-0.35,-0.21) | 0.000 | -0.18(-0.25,-0.11) | 0.000 |
| Age | -0.05(-0.05,-0.04) | 0.000 | -0.03(-0.04,-0.03) | 0.000 | -0.02(-0.03,-0.02) | 0.000 |
| Female | -0.94(-1.01,-0.86) | 0.000 | -0.45(-0.55,-0.36) | 0.000 | -0.50(-0.60,-0.41) | 0.000 |
| Rural |  |  | -0.34(-0.42,-0.26) | 0.000 | -0.21(-0.28,-0.13) | 0.000 |
| Widow/div/Sep/others | | | -0.19(-0.26,-0.13) | 0.000 | -0.15(-0.21,-0.09) | 0.000 |
| Caste (ref: none) | | |  |  |  |  |
| SCs/STs |  |  | -0.21(-0.29,-0.12) | 0.000 | -0.16(-0.24,-0.07) | 0.001 |
| OBCs |  |  | -0.08(-0.17,0.02) | 0.129 | -0.06(-0.16,0.05) | 0.279 |
| Religion (ref: Hindu) | | |  |  |  |  |
| Muslim |  |  | 0.00(-0.12,0.11) | 0.938 | -0.04(-0.17,0.10) | 0.594 |
| Others |  |  | 0.22(0.06,0.37) | 0.008 | 0.12(-0.02,0.26) | 0.102 |
| Education (ref: no schooling) | | |  |  |  |  |
| Standard 1-4 | |  | 0.76(0.61,0.90) | 0.000 | 0.67(0.53,0.81) | 0.000 |
| Standard 5-9 | |  | 1.19(1.03,1.34) | 0.000 | 1.03(0.88,1.18) | 0.000 |
| Standard 10 and above | |  | 1.37(1.21,1.53) | 0.000 | 1.07(0.91,1.23) | 0.000 |
| MPCE quintile (ref: poorest) | | |  |  |  |  |
| Poorer |  |  | 0.00(-0.08,0.09) | 0.910 | -0.02(-0.10,0.05) | 0.544 |
| Middle |  |  | 0.05(-0.05,0.15) | 0.317 | -0.01(-0.11,0.09) | 0.836 |
| Richer |  |  | 0.07(-0.05,0.19) | 0.249 | -0.01(-0.12,0.11) | 0.910 |
| Richest |  |  | 0.10(-0.01,0.22) | 0.075 | 0.01(-0.09,0.11) | 0.900 |
| Working status (ref: working) | | | |  |  |  |
| Not working | |  | -0.04(-0.19,0.12) | 0.647 | -0.02(-0.17,0.14) | 0.841 |
| Working |  |  | 0.06(-0.10,0.22) | 0.460 | 0.01(-0.16,0.17) | 0.943 |
| Smoking: no | |  |  |  | 0.00(-0.08,0.07) | 0.983 |
| Drinking: no | |  |  |  | 0.18(0.07,0.29) | 0.003 |
| Physical activity | |  |  |  | 0.02(0.01,0.03) | 0.000 |
| Social activity | |  |  |  | 0.02(0.01,0.02) | 0.000 |
| BMI |  |  |  |  | 0.02(0.02,0.03) | 0.000 |
| SRH (ref: good/very good) | | |  |  |  |  |
| Fair |  |  |  |  | 0.01(-0.07,0.09) | 0.837 |
| Poor/very poor | |  |  |  | -0.12(-0.25,0.00) | 0.052 |
| ADL limitations | |  |  |  | -0.19(-0.27,-0.11) | 0.000 |
| IADL limitations | |  |  |  | -0.24(-0.34,-0.14) | 0.000 |
| Chronic disease: yes | |  |  |  | 0.03(-0.01,0.08) | 0.168 |
| Childhood SRH (ref: good/very good) | | | |  |  |  |
| Fair |  |  |  |  | -0.06(-0.17,0.04) | 0.230 |
| Poor/very poor | |  |  |  | -0.05(-0.22,0.12) | 0.559 |
| Childhood finance (ref: financially well) | | | |  |  |  |
| Average |  |  |  |  | 0.04(-0.05,0.12) | 0.388 |
| Poor |  |  |  |  | -0.03(-0.17,0.11) | 0.667 |

| **Table S7: Associations between the level of food insecurity and arithmetic function among older adults, LASI wave 1, 2017-18** | | | | | | |
| --- | --- | --- | --- | --- | --- | --- |
|  | **Model I** | | **Model-II** | | **Model-III** | |
|  | **β (95%CI)** | **p-value** | **β (95%CI)** | **p-value** | **β (95%CI)** | **p-value** |
| Food insecurity (ref: food secure) | | | |  |  |  |
| Mild food  insecure | -0.22(-0.29,-0.15) | 0.000 | -0.08(-0.14,-0.03) | 0.002 | -0.06(-0.11,-0.01) | 0.027 |
| Moderate  food insecure | -0.67(-0.90,-0.44) | 0.000 | -0.19(-0.37,-0.01) | 0.036 | -0.11(-0.29,0.06) | 0.202 |
| Severe  food insecure | -0.95(-1.10,-0.80) | 0.000 | -0.20(-0.32,-0.08) | 0.001 | -0.09(-0.20,0.03) | 0.153 |
| Age | -0.07(-0.08,-0.06) | 0.000 | -0.04(-0.05,-0.04) | 0.000 | -0.03(-0.04,-0.03) | 0.000 |
| Female | -2.47(-2.72,-2.23) | 0.000 | -1.16(-1.33,-0.99) | 0.000 | -1.19(-1.38,-1.01) | 0.000 |
| Rural |  |  | -0.56(-0.73,-0.40) | 0.000 | -0.33(-0.48,-0.17) | 0.000 |
| Widow/div/Sep/others | | | -0.12(-0.22,-0.03) | 0.000 | -0.07(-0.16,0.03) | 0.000 |
| Caste (ref: none) | | |  |  |  |  |
| SCs/STs |  |  | -0.51(-0.71,-0.32) | 0.000 | -0.42(-0.62,-0.22) | 0.001 |
| OBCs |  |  | 0.02(-0.20,0.24) | 0.129 | 0.06(-0.14,0.26) | 0.279 |
| Religion (ref: Hindu) | | |  |  |  |  |
| Muslim |  |  | 0.12(-0.09,0.33) | 0.938 | 0.09(-0.16,0.34) | 0.594 |
| Others |  |  | -0.04(-0.35,0.28) | 0.008 | -0.18(-0.51,0.14) | 0.102 |
| Education (ref: no schooling) | | |  |  |  |  |
| Standard 1-4 | |  | 1.85(1.66,2.03) | 0.000 | 1.68(1.49,1.87) | 0.000 |
| Standard 5-9 | |  | 3.04(2.88,3.20) | 0.000 | 2.78(2.61,2.95) | 0.000 |
| Standard 10 and above | |  | 4.02(3.84,4.20) | 0.000 | 3.54(3.33,3.74) | 0.000 |
| MPCE quintile (ref: poorest) | | |  |  |  |  |
| Poorer |  |  | 0.16(0.07,0.26) | 0.910 | 0.10(0.01,0.20) | 0.544 |
| Middle |  |  | 0.31(0.16,0.47) | 0.317 | 0.20(0.05,0.35) | 0.836 |
| Richer |  |  | 0.42(0.24,0.59) | 0.249 | 0.25(0.09,0.42) | 0.910 |
| Richest |  |  | 0.53(0.36,0.69) | 0.075 | 0.31(0.15,0.47) | 0.900 |
| Working status (ref:working) | | | |  |  |  |
| Not working | |  | 0.20(0.05,0.36) | 0.647 | 0.20(0.07,0.34) | 0.841 |
| Working |  |  | 0.40(0.22,0.58) | 0.460 | 0.32(0.16,0.49) | 0.943 |
| Smoking: no | |  |  |  | -0.11(-0.24,0.03) | 0.983 |
| Drinking: no | |  |  |  | 0.04(-0.12,0.20) | 0.003 |
| Physical activity | |  |  |  | 0.04(0.02,0.06) | 0.000 |
| Social activity | |  |  |  | 0.04(0.02,0.05) | 0.000 |
| BMI |  |  |  |  | 0.05(0.03,0.06) | 0.000 |
| SRH (ref: good/very good) | | |  |  |  |  |
| Fair |  |  |  |  | 0.04(-0.07,0.16) | 0.837 |
| Poor/very poor | |  |  |  | 0.04(-0.17,0.24) | 0.052 |
| ADL limitations | |  |  |  | -0.21(-0.31,-0.11) | 0.000 |
| IADL limitations | |  |  |  | -0.14(-0.30,0.01) | 0.000 |
| Chronic disease: yes | |  |  |  | 0.13(0.03,0.22) | 0.168 |
| Childhood SRH (ref: good/very good) | | | |  |  |  |
| Fair |  |  |  |  | -0.16(-0.30,-0.02) | 0.230 |
| Poor/very poor | |  |  |  | 0.21(0.01,0.41) | 0.559 |
| Childhood finance (ref: financially well) | | | |  |  |  |
| Average |  |  |  |  | 0.04(-0.05,0.13) | 0.388 |
| Poor |  |  |  |  | -0.12(-0.26,0.02) | 0.667 |

| **Table S8: Associations between the level of food insecurity and executive function among older adults, LASI wave 1, 2017-18** | | | | | | |
| --- | --- | --- | --- | --- | --- | --- |
|  | **Model I** | | **Model-II** | | **Model-III** | |
|  | **β (95%CI)** | **p-value** | **β (95%CI)** | **p-value** | **β (95%CI)** | **p-value** |
| Food insecurity (ref: food secure) | | | |  |  |  |
| Mild food insecure | -0.22(-0.29,-0.15) | 0.000 | -0.08(-0.14,-0.03) | 0.002 | -0.06(-0.11,-0.01) | 0.027 |
| Moderate  food insecure | -0.67(-0.90,-0.44) | 0.000 | -0.19(-0.37,-0.01) | 0.036 | -0.11(-0.29,0.06) | 0.202 |
| Severe food insecure | -0.95(-1.10,-0.80) | 0.000 | -0.20(-0.32,-0.08) | 0.001 | -0.09(-0.20,0.03) | 0.153 |
| Age | -0.02(-0.03,-0.02) | 0.000 | -0.02(-0.02,-0.01) | 0.000 | -0.01(-0.02,-0.01) | 0.000 |
| Female | -0.43(-0.49,-0.37) | 0.000 | -0.11(-0.17,-0.06) | 0.000 | -0.10(-0.17,-0.04) | 0.000 |
| Rural |  |  | -0.17(-0.25,-0.09) | 0.000 | -0.10(-0.17,-0.02) | 0.000 |
| Widow/div/Sep/others | | | -0.07(-0.11,-0.03) | 0.000 | -0.04(-0.08,-0.01) | 0.000 |
| Caste (ref: none) | | |  |  |  |  |
| SCs/STs |  |  | -0.04(-0.12,0.03) | 0.000 | -0.03(-0.11,0.05) | 0.001 |
| OBCs |  |  | 0.03(-0.04,0.09) | 0.129 | 0.04(-0.02,0.10) | 0.279 |
| Religion (ref: Hindu) | | |  |  |  |  |
| Muslim |  |  | -0.05(-0.19,0.08) | 0.938 | -0.04(-0.18,0.10) | 0.594 |
| Others |  |  | -0.13(-0.23,-0.03) | 0.008 | -0.18(-0.28,-0.08) | 0.102 |
| Education (ref: no schooling) | | |  |  |  |  |
| Standard 1-4 | |  | 0.36(0.28,0.44) | 0.000 | 0.30(0.22,0.38) | 0.000 |
| Standard 5-9 | |  | 0.59(0.52,0.67) | 0.000 | 0.50(0.42,0.58) | 0.000 |
| Standard 10 and above | |  | 0.94(0.85,1.04) | 0.000 | 0.76(0.66,0.86) | 0.000 |
| MPCE quintile (ref: poorest) | | |  |  |  |  |
| Poorer |  |  | 0.05(0.00,0.10) | 0.910 | 0.03(-0.02,0.08) | 0.544 |
| Middle |  |  | 0.09(0.03,0.15) | 0.317 | 0.05(-0.01,0.12) | 0.836 |
| Richer |  |  | 0.11(0.03,0.18) | 0.249 | 0.06(-0.02,0.13) | 0.910 |
| Richest |  |  | 0.12(0.02,0.22) | 0.075 | 0.06(-0.04,0.16) | 0.900 |
| Working status (ref:working) | | | |  |  |  |
| Not working | |  | 0.10(0.01,0.18) | 0.647 | 0.09(0.02,0.17) | 0.841 |
| Working |  |  | 0.14(0.06,0.22) | 0.460 | 0.08(0.01,0.15) | 0.943 |
| Smoking: no | |  |  |  | 0.00(-0.05,0.06) | 0.983 |
| Drinking: no | |  |  |  | -0.06(-0.16,0.04) | 0.003 |
| Physical activity | |  |  |  | 0.02(0.01,0.03) | 0.000 |
| Social activity | |  |  |  | 0.01(0.01,0.02) | 0.000 |
| BMI |  |  |  |  | 0.01(0.00,0.01) | 0.000 |
| SRH (ref: good/very good) | | |  |  |  |  |
| Fair |  |  |  |  | -0.01(-0.08,0.06) | 0.837 |
| Poor/very poor | |  |  |  | -0.07(-0.16,0.02) | 0.052 |
| ADL limitations | |  |  |  | -0.07(-0.14,0.00) | 0.000 |
| IADL limitations | |  |  |  | -0.10(-0.17,-0.02) | 0.000 |
| Chronic disease: yes | |  |  |  | 0.02(-0.02,0.07) | 0.168 |
| Childhood SRH (ref: good/very good) | | | |  |  |  |
| Fair |  |  |  |  | -0.12(-0.20,-0.03) | 0.230 |
| Poor/very poor | |  |  |  | -0.10(-0.19,0.00) | 0.559 |
| Childhood finance (ref: financially well) | | | |  |  |  |
| Average |  |  |  |  | -0.12(-0.21,-0.03) | 0.388 |
| Poor |  |  |  |  | -0.13(-0.22,-0.04) | 0.667 |

| **Table S9: Associations between the level of food insecurity and object naming among older adults, LASI wave 1, 2017-18** | | | | | | |
| --- | --- | --- | --- | --- | --- | --- |
|  | **Model I** | | **Model-II** | | **Model-III** | |
|  | **β (95%CI)** | **p-value** | **β (95%CI)** | **p-value** | **β (95%CI)** | **p-value** |
| Food insecurity (ref: food secure) | | | |  |  |  |
| Mild food  insecure | -0.03(-0.04,-0.02) | 0.000 | -0.03(-0.04,-0.02) | 0.000 | -0.03(-0.03,-0.02) | 0.000 |
| Moderate  food insecure | -0.04(-0.06,-0.01) | 0.008 | -0.03(-0.06,0.00) | 0.027 | -0.02(-0.05,0.01) | 0.117 |
| Severe  food insecure | -0.05(-0.07,-0.04) | 0.000 | -0.04(-0.06,-0.03) | 0.000 | -0.03(-0.05,-0.02) | 0.000 |
| Age | 0.00(-0.01,0.00) | 0.000 | 0.00(0.00,0.00) | 0.000 | 0.00(0.00,0.00) | 0.000 |
| Female | -0.03(-0.04,-0.02) | 0.000 | 0.00(-0.01,0.01) | 0.000 | 0.00(-0.01,0.02) | 0.000 |
| Rural |  |  | -0.01(-0.02,0.00) | 0.000 | 0.00(-0.01,0.01) | 0.000 |
| Widow/div/Sep/others | | | -0.01(-0.03,0.00) | 0.000 | -0.01(-0.02,0.00) | 0.000 |
| Caste (ref: none) | | |  |  |  |  |
| SCs/STs |  |  | -0.02(-0.04,-0.01) | 0.000 | -0.02(-0.03,0.00) | 0.001 |
| OBCs |  |  | -0.01(-0.02,0.01) | 0.129 | -0.01(-0.02,0.01) | 0.279 |
| Religion (ref: Hindu) | | |  |  |  |  |
| Muslim |  |  | -0.01(-0.02,0.01) | 0.938 | -0.01(-0.02,0.01) | 0.594 |
| Others |  |  | -0.03(-0.06,-0.01) | 0.008 | -0.04(-0.06,-0.01) | 0.102 |
| Education (ref: no schooling) | | |  |  |  |  |
| Standard 1-4 | |  | 0.03(0.01,0.05) | 0.000 | 0.02(0.01,0.04) | 0.000 |
| Standard 5-9 | |  | 0.03(0.02,0.05) | 0.000 | 0.02(0.01,0.04) | 0.000 |
| Standard 10 and above | |  | 0.03(0.02,0.05) | 0.000 | 0.01(-0.01,0.03) | 0.000 |
| MPCE quintile (ref: poorest) | | |  |  |  |  |
| Poorer |  |  | 0.01(0.00,0.02) | 0.910 | 0.01(-0.01,0.02) | 0.544 |
| Middle |  |  | 0.02(0.00,0.03) | 0.317 | 0.01(0.00,0.03) | 0.836 |
| Richer |  |  | 0.02(0.00,0.04) | 0.249 | 0.01(0.00,0.03) | 0.910 |
| Richest |  |  | 0.01(-0.01,0.03) | 0.075 | 0.01(-0.01,0.02) | 0.900 |
| Working status (ref:working) | | | |  |  |  |
| Not working | |  | 0.03(0.01,0.05) | 0.647 | 0.03(0.01,0.05) | 0.841 |
| Working |  |  | 0.04(0.03,0.06) | 0.460 | 0.04(0.02,0.06) | 0.943 |
| Smoking: no | |  |  |  | -0.01(-0.02,0.01) | 0.983 |
| Drinking: no | |  |  |  | -0.01(-0.02,0.01) | 0.003 |
| Physical activity | |  |  |  | 0.00(0.00,0.00) | 0.000 |
| Social activity | |  |  |  | 0.00(0.00,0.00) | 0.000 |
| BMI |  |  |  |  | 0.00(0.00,0.00) | 0.000 |
| SRH (ref: good/very good) | | |  |  |  |  |
| Fair |  |  |  |  | 0.02(0.00,0.03) | 0.837 |
| Poor/very poor | |  |  |  | 0.00(-0.02,0.03) | 0.052 |
| ADL limitations | |  |  |  | -0.03(-0.05,-0.01) | 0.000 |
| IADL limitations | |  |  |  | -0.02(-0.03,0.00) | 0.000 |
| Chronic disease: yes | |  |  |  | 0.02(0.01,0.03) | 0.168 |
| Childhood SRH (ref: good/very good) | | | |  |  |  |
| Fair |  |  |  |  | -0.04(-0.06,-0.02) | 0.230 |
| Poor/very poor | |  |  |  | -0.05(-0.10,-0.01) | 0.559 |
| Childhood finance (ref: financially well) | | | |  |  |  |
| Average |  |  |  |  | -0.02(-0.03,0.00) | 0.388 |
| Poor |  |  |  |  | -0.02(-0.03,0.00) | 0.667 |

| **Table S10: Associations between the level of food insecurity and cognition among older adults, LASI wave 1, 2017-18** | | | | | | |
| --- | --- | --- | --- | --- | --- | --- |
|  | **Model I** | | **Model-II** | | **Model-III** | |
|  | **β (95%CI)** | **p-value** | **β (95%CI)** | **p-value** | **β (95%CI)** | **p-value** |
| Food insecurity (ref: food secure) | | | |  |  |  |
| Mild food  insecure | -0.57(-0.74,-0.40) | 0.000 | -0.27(-0.41,-0.13) | 0.000 | -0.18(-0.32,-0.04) | 0.010 |
| Moderate  food insecure | -1.60(-2.17,-1.03) | 0.000 | -0.48(-0.95,-0.01) | 0.044 | -0.13(-0.59,0.32) | 0.563 |
| Severe  food insecure | -2.81(-3.19,-2.44) | 0.000 | -1.05(-1.36,-0.74) | 0.000 | -0.52(-0.82,-0.21) | 0.001 |
| Age | -0.26(-0.28,-0.23) | 0.000 | -0.19(-0.20,-0.17) | 0.000 | -0.14(-0.16,-0.12) | 0.000 |
| Female | -4.73(-5.13,-4.32) | 0.000 | -1.82(-2.17,-1.47) | 0.000 | -2.06(-2.45,-1.68) | 0.000 |
| Rural |  |  | -1.71(-2.22,-1.20) | 0.000 | -0.93(-1.41,-0.45) | 0.000 |
| Widow/div/Sep/others | | | -0.75(-1.01,-0.49) | 0.000 | -0.52(-0.78,-0.26) | 0.000 |
| Caste (ref: none) | | |  |  |  |  |
| SCs/STs |  |  | -1.19(-1.71,-0.68) | 0.000 | -0.89(-1.43,-0.35) | 0.001 |
| OBCs |  |  | 0.02(-0.52,0.57) | 0.129 | 0.15(-0.38,0.67) | 0.279 |
| Religion (ref: Hindu) | | |  |  |  |  |
| Muslim |  |  | 0.08(-0.52,0.69) | 0.938 | -0.03(-0.79,0.74) | 0.594 |
| Others |  |  | 0.01(-0.65,0.68) | 0.008 | -0.54(-1.27,0.20) | 0.102 |
| Education (ref: no schooling) | | |  |  |  |  |
| Standard 1-4 | |  | 3.68(3.20,4.17) | 0.000 | 3.15(2.66,3.64) | 0.000 |
| Standard 5-9 | |  | 6.21(5.81,6.62) | 0.000 | 5.32(4.92,5.71) | 0.000 |
| Standard 10 and above | |  | 8.90(8.45,9.36) | 0.000 | 7.14(6.70,7.58) | 0.000 |
| MPCE quintile (ref: poorest) | | |  |  |  |  |
| Poorer |  |  | 0.28(0.01,0.56) | 0.910 | 0.09(-0.16,0.34) | 0.544 |
| Middle |  |  | 0.72(0.28,1.16) | 0.317 | 0.33(-0.08,0.74) | 0.836 |
| Richer |  |  | 0.96(0.46,1.47) | 0.249 | 0.46(0.00,0.92) | 0.910 |
| Richest |  |  | 1.29(0.74,1.85) | 0.075 | 0.63(0.15,1.10) | 0.900 |
| Working status (ref: working) | | | |  |  |  |
| Not working | |  | 0.24(-0.22,0.71) | 0.647 | 0.29(-0.13,0.70) | 0.841 |
| Working |  |  | 0.74(0.21,1.27) | 0.460 | 0.36(-0.14,0.86) | 0.943 |
| Smoking: no | |  |  |  | -0.13(-0.47,0.22) | 0.983 |
| Drinking: no | |  |  |  | 0.60(0.15,1.05) | 0.003 |
| Physical activity | |  |  |  | 0.16(0.12,0.21) | 0.000 |
| Social activity | |  |  |  | 0.12(0.09,0.15) | 0.000 |
| BMI |  |  |  |  | 0.13(0.10,0.16) | 0.000 |
| SRH (ref: good/very good) | | |  |  |  |  |
| Fair |  |  |  |  | -0.07(-0.44,0.31) | 0.837 |
| Poor/very poor | |  |  |  | -0.66(-1.19,-0.14) | 0.052 |
| ADL limitations | |  |  |  | -0.82(-1.11,-0.52) | 0.000 |
| IADL limitations | |  |  |  | -0.73(-1.10,-0.36) | 0.000 |
| Chronic disease: yes | |  |  |  | 0.28(0.00,0.57) | 0.168 |
| Childhood SRH (ref: good/very good) | | | |  |  |  |
| Fair |  |  |  |  | -0.44(-0.77,-0.12) | 0.230 |
| Poor/very poor | |  |  |  | 0.20(-0.42,0.81) | 0.559 |
| Childhood finance (ref: financially well) | | | |  |  |  |
| Average |  |  |  |  | 0.11(-0.21,0.43) | 0.388 |
| Poor |  |  |  |  | -0.48(-0.86,-0.09) | 0.667 |
